# Whole-exome sequencing analysis identifies novel variants associated with Kawasaki disease susceptibility

**DOI:** 10.1186/s12969-023-00857-0

**Published:** 2023-08-07

**Authors:** Xing Zhang, Ying Sun, Lijuan Meng, Caixia Ye, Huifeng Han, Tiesong Zhang, Yue Feng, Jianxiao Li, Lifen Duan, Yanfei Chen

**Affiliations:** 1 Department of Cardiology, Kunming Children’s Hospital, Yunnan Province Clinical Research Center for Children’s Health and Disease, Yunnan, China; 2 Maternity and Child health care Hospital of Yunyang County, Chongqing, China; 3 Capital Normal University, Beijing, China; 4 Kunming University of Science and Technology, Kunming, China

**Keywords:** Kawasaki disease, Susceptibility, Whole-exome sequencing, Association study

## Abstract

**Background:**

Kawasaki disease (KD) is an acute pediatric vasculitis affecting genetically susceptible infants and children. Although the pathogenesis of KD remains unclear, growing evidence links genetic susceptibility to the disease.

**Methods:**

To explore the genes associated with susceptibility in KD, we applied whole-exome sequencing to KD and control subjects from Yunnan province, China. We conducted association study analysis on the two groups.

**Results:**

In this study, we successfully identified 11 significant rare variants in two genes (*MYH14* and *RBP3*) through the genotype/allele frequency analysis. A heterozygous variant (c.2650G > A, p.V884M) of the RBP3 gene was identified in 12 KD cases, while eight heterozygous variants (c.566G > A, p.R189H; c.1109 C > T, p.S370L; c.3917T > G, p.L1306R; c.4301G > A, p.R1434Q; c.5026 C > T, p.R1676W; c.5329 C > T, p.R1777C; c.5393 C > A, p.A1798D and c.5476 C > T, p.R1826C) of the MYH14 gene were identified in 8 KD cases respectively.

**Conclusion:**

This study suggested that nine variants in *MYH14* and *RBP3* gene may be associated with KD susceptibility in the population from Yunnan province.

**Supplementary Information:**

The online version contains supplementary material available at 10.1186/s12969-023-00857-0.

## Introduction

Kawasaki disease (KD; OMIM611775) is an acute, self-limiting systemic vasculitis syndrome with the main clinical manifestations of fever, oral mucosal changes, rash, cervical lymphadenopathy, conjunctivitisin, and extremity changes, known as mucocutaneous lymph node syndrome (MCLS) [1, 2]. It was first described by Japanese pediatrician Tomisaku Kawasaki and particularly affects children under five years of age. With an almost worldwide increase in incidence, KD becomes now the leading cause of acquired heart disease in children, as it may cause coronary artery lesions in 15–25% of untreated patients or in 5–10% of patients treated with intravenous immunoglobulin (IVIG) [3]. The incidence of incomplete KD, which accounts for 15% and 47% of all KD cases [4], has also been reported to be increasing, posing a threat to the health of children’s coronary arteries [5–7].

Although KD can be diagnosed based on its typical features ( such as fever, conjunctivitis, skin rashes, increased fibrinogen, etc.)[8], while the immunopathogenic mechanisms of this disease remain unclear. Common and rare genetic variants could form many complex traits with complex interactions [9–11]. Domestic and foreign studies have found that inflammation-related genes *IL-18* and *IL-1B* [1, 12, 13], *inositol 1, 4, 5-trisphosphate 3-kinase C (ITPKC)* [14, 15], and other gene polymorphisms are associated with KD [16]. Meanwhile, the family aggregation of KD patients indicated that genetic factors play an important role in the occurrence of KD [17–19]. However, the susceptibility loci obtained by the candidate gene method have been controversial because the results of various studies cannot lead to more accurate and consistent conclusions due to differences in race, environment, and sample content. Finding susceptibility genes associated with complex diseases at the genome-wide level is an effective approach to investigating polygenic diseases. Commonly used methods include genome-wide linkage studies, genome-wide association studies (GWAS), and whole-exome sequencing (WES) [20]. Most GWAS-derived Single nucleotide polymorphisms (SNPs) do not directly affect disease characteristics, but are an index marker linking disease-specific imbalances and pathogenic variants [16, 21, 22]. Therefore, it is necessary to use other methods to identify rare coding variants that affect KD susceptibility. WES is one of the efficient sequencing techniques to identify rare protein-coding variants. In this study, we determined to identify the KD-associated protein-coding variants through WES that may provide new insights into diagnosis and treatment of KD.

## Materials and methods

### Patients and samples

The case-control sample set used in this study included 93 KD patients and 91 non-KD control cases. All cases were obtained from the Kunming Children’s Hospital, Yunnan province, southern China, and unrelated to each other. Inclusion criteria for KD cases included patients met the criteria for KD (fever together with principal symptoms such as conjunctivitis, skin rashes, increased fibrinogen, etc.)[8, 23], non-infectious and no previous cancer or metastases. The control cases were non-KD patients without fever. This study was approved by the Ethical Review Board of Kunming Children’s Hospital, and informed consent was obtained from all of KD and control subjects.

### Whole-exome sequencing and Sanger sequencing

WES was conducted using genomic DNA samples obtained from 93 children with KD. The exome sequences were efficiently enriched from 1 µg genomic DNA extracted from the peripheral blood using Agilent liquid capture system (Agilent Sure Select Human All Exon V6 kit, Agilent Technologies, Santa Clara, CA, USA) according to the manufacturer’s protocol. Finally, Illumina Novaseq 6000 platform (Illumina, San Diego, CA, USA), with 150 bp pair fragments sequencing mode, was used for sequencing the genomic DNA for shotgun library construction. The overall genotyping success rate was 99.5%. Raw image files were processed using CASAVA v1.82 for base calling and generating raw data.

Sanger sequencing was performed to confirm the variants identified by WES. PCR was conducted with TaKaRa Taq (Takara, Osaka, Japan) under the following conditions: 95 °C for 5 min; followed by 34 cycles at 95 °C for 30 s, 59 °C for 30 s and 72 °C for 30 s; 72 °C for 5 min. PCR products were purified by gel electrophoresis and sequenced using ABI 3730xl DNA Analyzer with the BigDye™ Terminator Cycle Sequencing Kit (Applied Biosystems, Foster, CA, USA).

### Statistical analysis

Variation frequencies were described as proportions, and SNP allele frequency comparisons between cases and controls were analyzed by Fisher’s exact tests and odds ratios [19], and 95% confidence intervals (CIs) calculated by unconditional logistic regression were used to analyze the association between SNPs and KD susceptibility. Two-tailed *p-value* < 0.05 was considered statistically significant.

## Results

### Baseline characteristics

In the KD group, there were 87 classic KD patients (93.55%), 8 incomplete KD patients (8.6%) and 2 unresponsive to IVIG (2.2%). The mean age of KD group was 2.40 ± 1.84 years (ranged from 7 days to 12 years), and the male to female ratio was 1.7:1 (59/34). It was slightly higher than the ratio of 1.5: 1 of KD patients generally, *p-value* < 0.05 according to Fisher’s exact test. The proportion of KD patients with coronary artery aneurysms was 8.6% (8, 93). The mean age of control group was 2.50 ± 1.76 years (ranged from 3 months to 12 years), and the male to female ratio was 1.6:1 (56/35). In KD and control groups, the proportions of Han were 80.65% (75, 93) and 84.62% (77, 91) respectively. The proportions of ethnicity were 19.35% (18, 93) and 15.38% (14, 91) respectively. The detail information of the patients was in supplement file 1.

### Filtering of candidate variants

A total of 349,054 variants were identified from exome sequence data of 93 KD cases and 91 controls. The filtering steps including: filtering by MAF (MAF < 0.01), Picking of damage variants, case-control analysis. MAF filtering according to the population frequency databases include including 1000G(1000 Genome), ExAC(Exome Aggregation Consortium), and gnomAD (Genome Aggregation Database). Frameshift mutations, terminator mutations, splice mutations, and missense mutation with a Combined Annotation Dependent Depletion (CADD) [24] scores > 25 were retained. In the step, 8413 protein-altering variants remained, among which 6466 were missense SNV, 851 were frameshift, 394 were in splice-acceptor/donor sites, 687 were nonsense, and 15 were start lost.

### Associations with KD

**Fig. 1:**
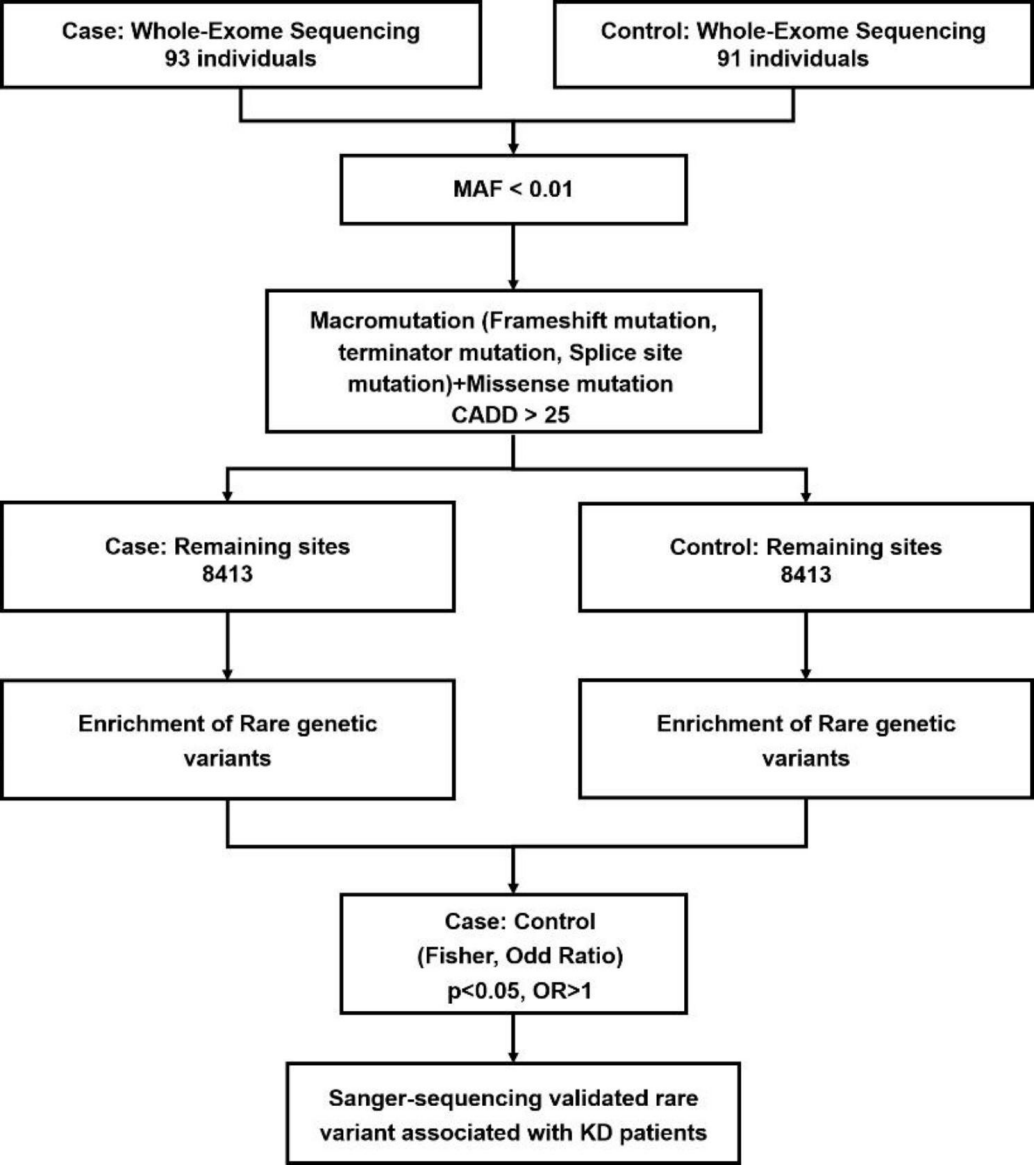
Overview of study design for data processing

ORs were valued by R package ggplot2, which performed deleterious when ORs > 1. Through association analysis of these 8413 variants, we successfully identified 11 variants in three genes (*TTI1*, *MYH14*, and *RBP3*) from 46 cases (Fig. 1; Table 1), which showed nominal significances (ORs = 2.3177 to 13.1963; *p* = 0.0025 to 0.0346) (Table 1). However, two variants in *TTI1* gene (appeared in 26 cases) were excluded as the high allele frequency in control group. A heterozygous variant (c.2650G > A, p.V884M) of the *RBP3* gene was identified in 12 KD cases, while eight heterozygous variants (c.566G > A, p.R189H; c.1109 C > T, p.S370L; c.3917T > G, p.L1306R; c.4301G > A, p.R1434Q; c.5026 C > T, p.R1676W; c.5329 C > T, p.R1777C; c.5393 C > A, p.A1798D and c.5476 C > T, p.R1826C) of the *MYH14* gene were identified in 8 KD cases respectively. All of the variants were confirmed by Sanger sequencing.

**Table 1:** Rare or frequency-unavailable variants associated with KD patients

Pt	Position	SNP ID	Gene	Exon	AA change	CDs	Accession	P Value	OR (95% CI)
4, 18, 22, 38, 47, 49, 56, 70, 73, 76, 84, 89	Chr10: 47,351,134	rs11204213	RBP3	Exon 1	p.V884M	c.2650G > A	NM_002900	0.0025^d^	13.1963(1.8761; 574.6005)
91	Chr19: 50,281,727	rs752792064	MYH14	Exon 31	p.R1434Q	c.4301G > A	NM_024729	0.0346^d^	8.3945(1.0870; 379.2676)
89	Chr19: 50,223,086	rs766546274		Exon 4	p.R189H	c.566G > A	NM_024729		
88	Chr19: 50,280,044	NA		Exon 29	p.L1306R	c.3917T > G	NM_024729		
88	Chr19: 50,301,707	rs368219210		Exon 38	p.A1798D	c.5393 C > A	NM_024729		
63	Chr19: 50,301,790	rs187789045		Exon 38	p.R1826C	c.5476 C > T	NM_024729		
30	Chr19: 50,293,670	rs377096949		Exon 37	p.R1777C	c.5329 C > T	NM_024729		
20	Chr19: 50,292,282	rs761720529		Exon 35	p.R1676W	c.5026 C > T	NM_024729		
6	Chr19: 50,244,260	rs150806988		Exon 10	p.S370L	c.1109 C > T	NM_024729		

Note: Pt, patients; SNP, single nucleotide polymorphism; CHR, chromosome; AA, amino acid; OR, odd ratio; NA, not available

*Bold values: The statistically significant (*p*-values < 0.05). ^d^Significant *p*-value

## Discussion

KD is one of the most common systemic vasculitic illness of children under the age of five years, leads to coronary artery aneurysms in 25% of untreated patients [8, 23]. It’s a multisystem inflammatory process, presumably, the etiology is an excessive immune response to possible infection or environmental triggers in genetically susceptible individuals [25]. People with KD may be inherently prone to other diseases, especially children younger than five years. Previous studies indicated that the incidence of KD in Asia was higher than that in the United States and Europe [25–29], and a higher incidence of males than females [2, 30]. Incidence within families is higher than in sporadic cases [17, 29]. KD could be regarded as a multifactorial and polygenic (complex) disorder [31, 32].

GWAS has identified some well-defined KD-associated loci and part of the genetic background successfully in recent studies, while it does not contribute significantly to exploring the pathogenesis of KD [33, 34]. Different from the GWAS, WES technology can explore global genetic mutations of many other complex diseases. It could discover rare mutations in the encoding sequence, which may cause its protein-coding variants that contribute to KD susceptibility. Jae-Jung Kim et al. explore the impact of coding variation on KD using WES for the first time [20], while no studies in the Chinese population.

In this study, we performed WES to identify rare protein-coding variants responsible for KD susceptibility. Nine variants in *RBP3* and *MYH14* gene were significantly enriched in KD cases. c.2650G > A (p.V884M) in exon 1 of *RBP3* gene were identified in 12 KD cases, appears to be present at a high rate (12.9%) in the KD group. Eight variants of the *MYH14* gene were identified in 8 KD cases respectively. All the allele frequencies were lower than 0.0275%, which indicated both of them were rare variants of genes. *RBP3* and *MYH14* gene are the first time reported to be associated with KD.

**Fig. 2:**
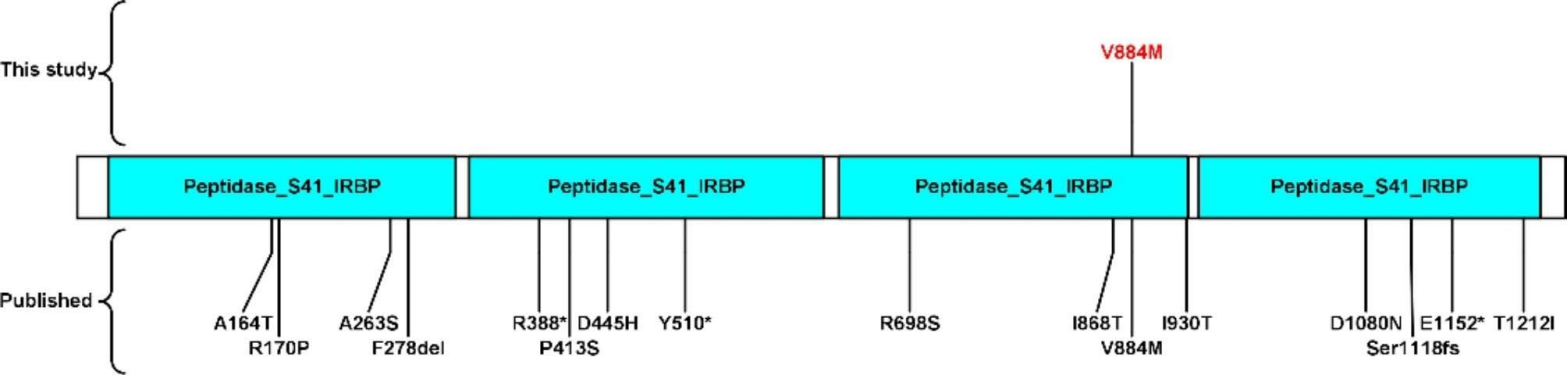
Protein diagrams shown for *RBP3* variants are depicted against a protein model. Variants (V884M) listed above the protein model are new to this study, while the ones below were published previously

*RBP3* gene encode interphotoreceptor retinol-binding protein, transport retinoids between the retinal pigment epithelium and the photoreceptors [35]. In 2015, Arno et al. first described retinal dystrophy in children caused by homozygous nonsense RBP3 mutations, highlighting the requirement for IRBP in normal eye development and visual function [36]. Yokomizo et al. [37] found that elevated expression of photoreceptor-secreted RBP3 may play a role in protection against the progression of diabetic retinopathy. To date, sixteen *RBP3* gene variants have been recorded in the HGMD database (https://www.hgmd.cf.ac.uk/ac/index.php), including eleven missense variants, three nonsense variants, one frameshift variant, and one fragment deletion variant (Fig. 2). In the study, c.2650G > A (p.V884M) locates in the third of four tandem homology modules [27, 38, 39], causes amino acid change from Valine to methionine. Chen P et al [27] indicated c.2650G > A (p.V884M) was associated with corneal curvature in Asian populations. Although previous researchers thought *RBP3* was associated with retinal retinoid transport and corneal changes, our research suggests an association with KD also. Conjunctivitis and subconjunctival hemorrhage are common phenotypes in KD [8, 23]. While in this research, c.2650G > A (p.V884M) maybe related to the ocular phenotypes in KD such as conjunctivitis and subconjunctival hemorrhage, which needs further research.

**Fig. 3:**
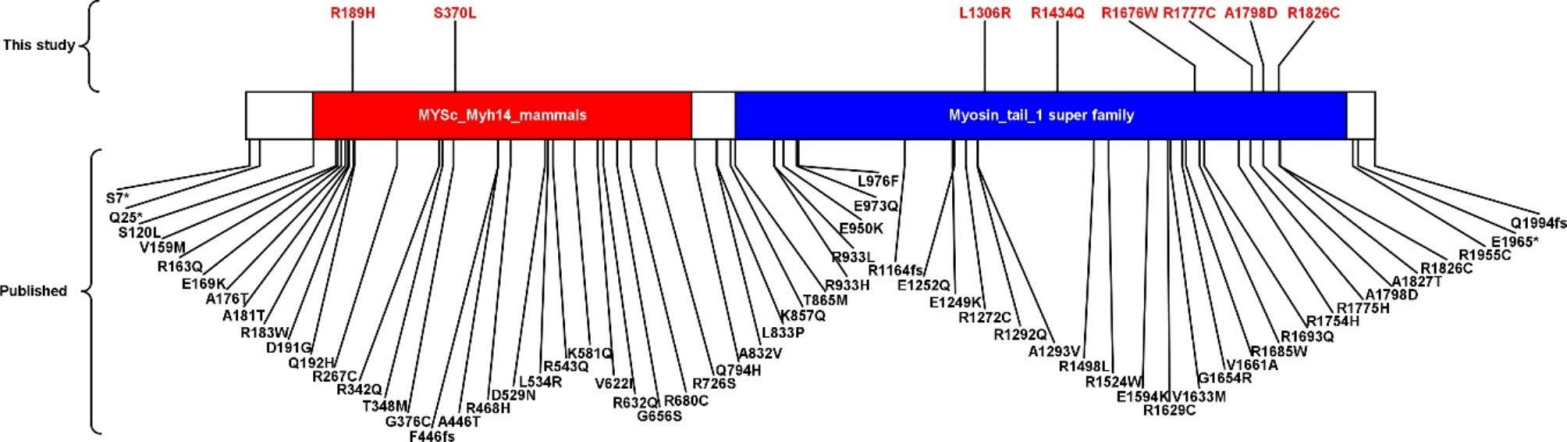
Protein diagrams shown for *MYH14* variants are depicted against a protein model. Variants (R189H, S370L, L1306R, R1434Q, R1676W, R1777C, A1798D, and R1826C) listed above the protein model are new to this study, while the ones below were published previously

*MYH14* is a member of the nonmuscle myosin II family of ATP-dependent molecular motors, which interact with cytoskeletal actin and regulate cytokinesis, cell motility, and cell polarity [40]. Sixty gene variants of *MYH14* gene have been recorded in the HGMD database, within 54 missense variants, three nonsense variants, and three frameshift variants. Missense variant is the most common pathogenic variant, scattered across the whole gene. Eight missense variants of *MYH1*4 gene were discovered in this study (Fig. 3), only c.5393 C > A( p.A1798D) has been published before. The homozygous variant (c.5393 C > A) may cause perineal fistulas in Anorectal malformations, based on the genetic and computer analyses, related to normal cloaca development by nonmuscle myosin heavy chain IIC (NMHC IIC) localization analysis [41]. It seems no significantly relevant to the result of this study. Wang M et al [42] indicated that c.5417 C > A (p.A1806D) in *MYH14* gene led to sensorineural hearing loss (SNHL). SNHL was reported in approximately 36% patients with Kawasaki [43], rare variants in *MYH14* gene maybe potentially associate with the symptom.

Above all, *RBP3* and *MYH14* were first reported to be associated with KD susceptibility in chinses population. This study’s limitation is a relatively small sample size, so the samples of coronary artery aneurysms and ethnic minorities were limited, unable to conduct more diversified data analysis. Studies on large sample sizes are needed in future to further reveal the relationship between candidate genes and KD.

## Conclusion

WES was performed on the KD and control group to identify susceptibility genes in patients from southwest China, and two protein-coding gene (*RBP3* and *MYH14*) were identified in the case-control analysis (ORs, 8.3945 to 13.1963; *p*-value, 0.0346 to 0.0025). These results provide insights into novel candidate genes and genetic variants that may be involved in KD and related KD complications. Further association studies with expanded KD samples from southwestern China or different ethnic groups are needed to confirm these results.

### Electronic supplementary material

Below is the link to the electronic supplementary material.

Supplementary Material 1

## Data Availability

All data generated or analyzed during this study are included in this published article and tables, and whole-exome sequencing analysis data has been uploaded to NCBI database (No. PRJNA869779).

## Abbreviations

KD: Kawasaki disease
MCLS: Mucocutaneous lymph node syndrome
ITPKC: *Inositol 1, 4, 5-trisphosphate 3-kinase C*
GWAS: Genome-wide association studies
WES: Whole-exome sequencing
Cis: Confidence intervals
MYH14: Myosin Heavy Chain 14
RBP3: Retinoid-binding protein 3
